# Hitting the Wall—Sensing and Signaling Pathways Involved in Plant Cell Wall Remodeling in Response to Abiotic Stress

**DOI:** 10.3390/plants7040089

**Published:** 2018-10-23

**Authors:** Lazar Novaković, Tingting Guo, Antony Bacic, Arun Sampathkumar, Kim L. Johnson

**Affiliations:** 1 School of BioSciences, University of Melbourne, Parkville, VIC 3010, Australia; tingtingg@student.unimelb.edu.au; 2 Max Planck Institute of Molecular Plant Physiology, 14476 Potsdam, Germany; Sampathkumar@mpimp-golm.mpg.de; 3 La Trobe Institute for Agriculture and Food, La Trobe University, Bundoora, VIC 3086, Australia; T.bacic@latrobe.edu.au

**Keywords:** cell wall, abiotic stress, signal transduction, reactive oxygen species, hormones, remodeling

## Abstract

Plant cells are surrounded by highly dynamic cell walls that play important roles regulating aspects of plant development. Recent advances in visualization and measurement of cell wall properties have enabled accumulation of new data about wall architecture and biomechanics. This has resulted in greater understanding of the dynamics of cell wall deposition and remodeling. The cell wall is the first line of defense against different adverse abiotic and biotic environmental influences. Different abiotic stress conditions such as salinity, drought, and frost trigger production of Reactive Oxygen Species (ROS) which act as important signaling molecules in stress activated cellular responses. Detection of ROS by still-elusive receptors triggers numerous signaling events that result in production of different protective compounds or even cell death, but most notably in stress-induced cell wall remodeling. This is mediated by different plant hormones, of which the most studied are jasmonic acid and brassinosteroids. In this review we highlight key factors involved in sensing, signal transduction, and response(s) to abiotic stress and how these mechanisms are related to cell wall-associated stress acclimatization. ROS, plant hormones, cell wall remodeling enzymes and different wall mechanosensors act coordinately during abiotic stress, resulting in abiotic stress wall acclimatization, enabling plants to survive adverse environmental conditions.

## 1. Introduction

Environmental stress (in short, stress) is defined as values of different environmental factors which are sub- or supra-optimal for survival of a given living system. Plants have developed efficient and variable mechanisms (morphological, anatomical, biochemical, and molecular) to acclimatize to different abiotic stresses. Stress can be classified in two broad groups: abiotic and biotic. Biotic stresses are caused by plant interactions with other organisms in the ecosystem, for example other plants of the same or different species, animals, fungi, and microorganisms (including parasitic interactions, grazing, allelopathic, and autopathic interactions) [1]. Biotic stresses are highly diverse, since plants engage in various interactions with many organisms in their environment. Responses to biotic stress have been well covered in many reviews [2,3,4] and will only briefly be addressed here. In this paper we will focus on the role of the cell wall in perception and response to different abiotic stresses. Abiotic stress is defined as nonliving factors in the environment that have a negative influence on plant growth and development. Among the best-studied responses are plant acclimatization strategies to drought [5,6,7,8,9], high soil salinity [10,11,12,13], cold stress [14,15,16,17], heat stress [18,19,20], and heavy metals [21,22,23]. In many cases these abiotic stresses affect the water equilibrium between the outside and inside of the cell that manifest as changes in turgor pressure. For example, during cold stress, freezing of water in the wall causes physical stress and dehydration. As the integrity of the wall is essential for generation of turgor, decreased water potential outside the cell can result in deformation of the wall and alterations in its contact with the plasma membrane. Loss of water can also cause enhanced bonding between wall polymers and influence the biosynthesis and deposition of new wall polymers. For example, during salt stress sodium ions can influence pectin cross-links and have also been shown to disrupt microtubule stability and therefore influence cellulose deposition [24]. Changes in the mechanical properties of the wall as a result of these changes as well as disruption of the contact between the wall and plasma membrane influence the function of cell wall integrity (CWI) sensors. Located at the plasma membrane CWI sensors transduce signals into the cytoplasm to initiate changes in the wall to maintain integrity and cellular function. While some excellent reviews have covered the response of the wall to abiotic stress [25,26], they have largely overlooked the important roles of wall integrity sensors to detect changes in wall mechanical properties during stress. In this paper we highlight recent developments in understanding the role of the wall in abiotic stress perception and the complex interplay between sensing and signaling pathways that ultimately remodel the wall.

In plants, the cell wall (in short, wall) is a complex polysaccharide-rich structure surrounding every plant cell. The continuum of walls throughout the plant body is known as the apoplast and is essential in water and nutrient uptake and transport, and is a site of post-deposition remodeling of wall functional properties [27]. There is a plethora of different walls with diverse structures, chemical compositions and mechanical properties depending on the function of the cell they encapsulate [28]. To exemplify how wall properties relate to function, irrespective of the cell type, protoplasted cells are all spherical and morphologically indistinguishable, whereas the thickened walls of vessel cells facilitate the transport of water, the mechanical anisotropy of guard cells enables pore opening, and the jigsaw patterns of pavement cells promote mechanical integrity. In addition to tissue level differences, distinct plant groups often display specific wall structures and composition [28,29,30,31]. The composition and architecture of the wall directs cell shape and influences the rate and direction of cell and tissue growth. These processes are controlled by complex and coordinated synthesis, disassembly, and remodeling of wall components [28,29]. The wall also has important roles in the perception of different environmental signals, resulting in activation of cellular responses to abiotic stress conditions [2,32]. Wall-mediated stress sensing and wall acclimatization are enabled by various receptors and channels that regulate transport, and exchange of local and long-distance molecular signals such as elicitors, hormones, sugars, proteins, and RNAs [2]. Wall-associated sensors have roles that include detection of mechanical signals generated by turgor pressure from inside the cell or from neighboring cells, and CWI sensors that detect changes in wall mechanical properties and activate signal transduction [33,34,35].

One of the earliest response to abiotic stress is either a considerable decrease or a complete cessation of plant growth [36]. Since growth is directed and regulated by wall synthesis and expansion, changes in the biomechanical properties of walls are an essential response to different stresses [37]. There are numerous challenges related to investigation of the role of the wall in stress acclimatization. It is important to note that different stress conditions are not acting independently on an individual plant, and plants in nature can be exposed to several different stresses at the same time [34,38]. All these considerations need to be taken into account when evaluating the effect of stress on plants as well as the fact that some mechanisms are used to fight both abiotic and biotic stress, further complicating studies of the role of the wall in abiotic stress acclimatization [34]. One of the challenges lies in fact that some of the cell systems used in wall-mediated stress sensing and acclimatization are active during both physiological and stress conditions (plant hormone signaling, ROS production, antioxidant enzymes, and cell wall integrity sensors) and it is challenging to make clear distinctions when these systems are involved in wall-mediated stress acclimatization and normal developmental processes.

Nevertheless, progress has been made and a number of studies have described the role of the wall in response to different abiotic stresses using either transcriptomic or proteomic approaches [39,40,41,42,43]. Recently, metabolomics-based studies of wall dynamics under abiotic stress have contributed to our knowledge of how adverse environmental conditions can affect wall composition and how this remodeling helps plants to acclimatize to stress [44,45,46,47]. In addition, molecular mechanisms of wall remodeling under stress conditions are being elucidated [48,49,50].

**Figure 1:**
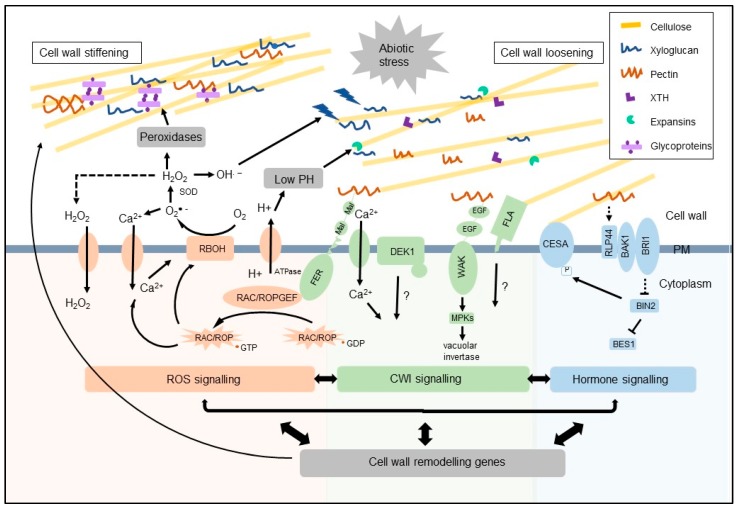
Model of the complex interactions between reactive oxidative species (ROS), cell wall integrity sensing (CWI), and hormone signaling pathways in a dicot primary wall during an abiotic stress. Abiotic stress can result in wall damage and changes in mechanical properties that activates ROS (orange), CWI sensing (green), and hormone signaling (blue). Respiratory burst oxidase homologues (RBOH) are activated by calcium (Ca^2+^) influx leading to the production of ROS (H_2_O_2_ and OH^●−^) in the wall. ROS production combined with peroxidase activity promotes oxidative cross-linking of extensins and signaling to induce pectate accumulation and wall stiffening. If peroxidase activity or H_2_O_2_ is limited, formation of OH^●−^ radicals could result in cleavage of sugar bonds in polysaccharides and wall loosening. CWI sensors from the CrRLK family, such as Feronia (FER), have been shown to interact with ROP-GEF to promote ROP2 GDP-GTP exchange and activation of RBOH and ROS production. FER is also proposed to activate H^+^-ATPases, potentially leading to increased extracellular pH and wall loosening. The extracellular domain of FER can interact with pectin and in response to stress-induced wall damage influences Ca^2+^ influx to maintain CWI. Defective Kernel 1 (DEK1) acts with a mechanosensitive Ca^2+^ channel and potentially regulates CWI during stress. Wall Associated Kinases (WAKs) are known to bind pectins and are proposed to initiate signaling via mitogen-activated protein kinases (MPKs) to regulate vacuolar invertases and turgor maintenance. Fasciclin-like arabinogalactan-proteins (FLAs) likely influence cellulose biosynthesis and/or deposition in response to stress. The negative regulator of brassinosteroid (BR) signaling, brassinosteroid insensitive 2 (BIN2), can phosphorylate cellulose synthase A (CESA) and reduce CESA activity. BR negatively regulates BIN2 to active the transcription factor BES1 and genes encoding cell wall loosening agents including xyloglucan endotransglycosidases/hydrolases (XETs/XTHs), expansins, and pectin modifying enzymes as well as genes involved in ROS production. RLP44 interacts with BR receptors BRI1 and BAK1 in response to wall damage and increases BR signaling. Fine-tuning the balance between these pathways regulates wall stiffening/loosening to modulate abiotic stress tolerance.

This review will focus on the role of the wall during abiotic stress sensing, signal transduction, and response. Wall-mediated responses to abiotic stresses involve generation of reactive oxygen species (ROS), plant hormone signaling [51], and cell wall integrity sensing [52] (Figure 1). Here, we highlight each of these networks and how they cooperatively interact to modulate the wall during abiotic stress conditions.

## 2. Plant Cell Walls

The walls of land plants are largely composed of three main types of polysaccharides; cellulose, non-cellulosic polysaccharides (NCPs, also, commonly referred to as “hemicelluloses”) and pectins. Some walls are also characterized by significant amounts of polyphenolic compounds [28]. Primary walls are laid down during cell division and in young, metabolically active, and growing tissues are sufficiently flexible to enable growth whilst maintaining mechanical strength to yield to (and withstand) turgor pressure. Secondary walls are laid down once cell expansion has ceased, are characterized by thickened walls with high mechanical strength and increased rigidity [28]. Polyphenolic compounds (such as lignin and its different derivatives) can constitute up to 30% of lignified secondary walls [53].

The major structural components of walls are the load-bearing cellulose microfibrils which are embedded in a matrix of NCPs and pectins. Whereas the cellulose microfibril network is common for all plant walls the composition of the matrix is highly variable and differs between different groups of plants and at different developmental stages (primary vs. secondary walls). In dicots, gymnosperms and noncommelinid monocots, the matrix of primary walls is composed primarily of xyloglucans and pectins [54]. Whereas primary walls of commelinid monocots have a high proportion of (glucurono)arabinoxylans and (1,3,1,4)-β-glucans with low levels of pectins and xyloglucans [54]. Proteins associated with the wall are highly diverse and include enzymes, receptors, and structural components [34,55]. Despite being a relatively minor component of the wall (approx. 10% (*w*/*w*) in primary walls but much less for secondary walls), these proteins can have an enormous influence on wall properties through modification of wall polysaccharides (and/or their interactions) and polyphenolics, as well as signaling functions.

Early plant wall models proposed that xyloglucans in dicot walls were the major matrix polysaccharides interacting with cellulose microfibrils [29]. More recent models suggest xyloglucan makes contact with cellulose at limited sites (“hot spots”) that are targeted by wall loosening proteins to enable expansion and rather it is pectins which make the majority of contacts with cellulose and these may be the primary mechanical tethers [56,57]. Pectins are involved in maintaining and controlling wall porosity by constituting a hydrated gel phase in which cellulose and NCPs are embedded. Pectin modifying enzymes, including pectin methylesterases (PMEs), pectin methylesterase inhibitors (PMEIs), and pectin degrading enzymes contribute to the complex regulation of pectin stiffness/viscosity that directly influences wall expansion and cell growth [58,59]. In addition, wall loosening proteins such as expansins and XETs/XTH, are crucial in modulating xyloglucan–cellulose interactions [58,59,60].

Wall composition and mechanical integrity are constantly monitored by cell wall integrity (CWI) sensors and mechanosensitive ion channels [33,61]. CWI sensors are essential for balancing wall strength and expansion during turgor driven growth. In addition to detecting changes in the physical properties of walls during normal growth and development [62,63,64], CWI sensors detect wall damage to cellulose and other polysaccharide components of the wall, such as pectins [65]. This damage may be caused by different abiotic and biotic stresses and results in wall remodeling to maintain mechanical integrity of the cell [66].

## 3. The Effect of Stress ‘Hitting’ the Wall

Although both abiotic and biotic stress results in wall damage, they act on different scales. Abiotic stresses are largely systemic (both symplastic and apolplastic) and entire organs are exposed to the adverse effects of stress. In contrast, biotic stress-induced wall damage is generally localized to the point of infection and adjacent areas. Biotic stress caused by pathogens results in wall decomposition caused by an arsenal of wall decomposing enzymes produced and secreted by pathogenic fungi and bacteria and include cellulases, hemicellulases, and pectinases which are also countered by the plant by the formation of localized appositions (termed papillae) in an attempt to “wall off” the invading pathogen [3]. Cell wall degradation induced by the pathogen is an important step in the infiltration process and the commensurate responses by the plant to rectify and reinforce the wall through specialized depositions of polysaccharides and polyphenolics is part of the defense dynamics [3,67].

Abiotic stresses, such as drought, heat, and cold (freezing), impact the water potential of the cell and therefore turgor pressure. In primary walls that are a highly hydrated (60–80% of their wet weight is water) gel-like matrix, this will influence both the organization and interaction of the wall polymers and also the physical attachment between the wall and the plasma membrane. Additionally, in the case of metal/ion toxicities, such as that of high soil salinity, monovalent ions such as Na^+^ and K^+^ can cause displacement of Ca^2+^ ions and disruption of the pectin “egg box” matrix structure. Thus, salt stress-induced changes in wall mechanical properties lead to a change of integrity that is perceived through the CWI sensors [68,69]. Nutrient deficiency, for example boron deficiency, causes severe developmental defects due to the role of boron in rhamnogalactouronan II (a pectic polysaccharide) cross-linking and their attachment to the cell membrane, causing increased wall thickness and a phenotype known as “swollen cell wall” [70,71]. Cold stress can lead to the formation of ice crystals in the apoplastic space. These crystals then cause rearrangement of cell wall architecture, which exert increased mechanical strain on the cell membrane, and in extreme cases, cell rupture [72,73].

Whilst some CWI sensors are involved in detecting wall damage induced by both abiotic and biotic stress, distinct sensors also exist. For example, pathogens are detected by specialized Receptor Like Kinases (RLKs) or Receptor Like Proteins (RLP) which can detect molecules originating from pathogens in the apoplast, called Pathogen Associated Molecular Patterns (PAMPs) [74]. PAMPs include chitin/chitin oligosaccharides from fungal walls, flagellin from bacterial flagellae, microbial metabolites, or even microbial enzymes [75]. These are detected by proteins such as lysine motif (Lys-M)-CHITIN ELICITOR RECEPTOR KINASE1 (OsCERK1) in *Oryza sativa* [76] and AtCERK1 in *A. thaliana*, which are recognizing chitin, or FLAGELLIN SENSING 2 (FLS2) which recognizes flagellin. In other cases, wall damage through Damage Associated Molecular Patterns (DAMPs) which are small fragments of plant wall produced by herbivore foraging [75] or microbial degradation [77] are detected. DAMP- and PAMP-related signaling triggers production of either plant secondary metabolites [78] or programmed cell death pathways [79] which is not generally the case during abiotic stress induced wall injury.

While signaling pathways of RLKs acting as CWI sensors involved in biotic stress responses are relatively well documented, their signaling during abiotic stress remains largely unknown [32]. In this paper, we outline some of the recent progress made in elucidating different CWI sensors signaling during abiotic stress. Some RLKs have been shown to act during both abiotic and biotic stress, such as BAK1. BAK1 can act as a co-receptor for BRASSINOSTEROID-INSENSITIVE 1 (BRI1) as well as many RLPs involved in PAMP and DAMP recognition [80]. BAK1 has also been shown to associate with a receptor-like protein RLP44 in response to pectin-induced changes in cell wall integrity [81] (Figure 1). There are numerous abiotic stress signaling pathways which are independent of biotic stress responses such as the Salt Overly Sensitive (SOS) pathways active under salt stress [82] and different signaling pathways invoked during heavy metal stress [83]. Some receptors are only responsive to abiotic stress such as *A. thaliana* Receptor Like Protein Kinase 1 (RPK1), which when overexpressed leads to increased tolerance to drought, heat, salt, and cold stress [84]. Another putative abiotic stress sensor that is possibly involved in cold stress acclimatization is COLD1 [85]. Although cold responsive genes have been shown to participate in plant acclimatization to cold stress [86], a connection with cell wall remodeling is yet to be shown.

Although wall acclimatization to abiotic and biotic stress can sometimes result in similar outcomes, the underlying signaling pathways likely differ and therefore require a greater knowledge of the pathways involved. One well documented plant response during both abiotic and biotic stress is ectopic lignin deposition [52]. Lignin is a component of many different types of secondary walls in plants, however here we will refer to lignin deposition only as one of mechanism of stress-induced wall remodeling. Another important response is wall glycoprotein cross-linking to promote wall stiffening.

This myriad of different changes in the organization of the wall, including changes in the biosynthesis of wall components caused by stress, are sensed by the CWI sensors to coordinate the cellular response and thereby enable stress-induced wall acclimatization. Wall acclimatization is one of the plant’s responses to abiotic stress and it enables plants to survive adverse environmental conditions. In ecophysiology, the definition of acclimatization is disputed, but in this paper, we will regard acclimatization as stress-induced changes in the organism’s metabolism and/or development (in this particular case, the wall), which increases the organisms chances of survival.

## 4. Role of Reactive Oxidative Species in Wall-Mediated Stress Response

ROS have an oxygen atom with an unpaired valence electron, the most common being hydrogen peroxide (H_2_O_2_), hydroxyl radical (OH^●−^), superoxide anion (O_2_^●−^), and nitric oxide (NO^–^). Historically ROS were considered toxic waste products of metabolism [87], the majority being generated either during the photochemical phase of photosynthesis, mitochondrial respiration, and photorespiration, or the result of damage to cellular components caused by different stress factors [88]. The high energy content of ROS enables them to quickly oxidize compounds, such as lipids, RNA, DNA, and polysaccharides rendering them nonfunctional or damaged. They can also initiate membrane oxidation, causing electron leakage, decreases in photosynthesis yield, degradation of cell organelles and cell death [88,89]. This has prompted intensive research into ROS detoxifying mechanisms, such as ascorbate-glutathione antioxidant systems, ROS-scavenging enzymes including ascorbate peroxidases (APXs), catalases (CATs), superoxide dismutases (SODs), peroxiporines, and others [89,90,91,92].

Many studies have shown the importance of ROS signals during the early stages of plant responses to both abiotic and biotic stresses [93] and ROS are viewed as important developmental regulators [94]. It is important to make the distinction between the role of ROS during biotic and abiotic stresses. In some cases, similar types of responses are activated for both abiotic and biotic stress [52,95] and, in other cases, ROS have distinct responses during biotic stress when compared with their function in abiotic stress. For example, during pathogen attack, plants produce ROS either to directly destroy pathogens or induce cell death and localized tissue necrosis by oxidative burst, or to stimulate transcription of different defense or pathogen immunity genes [95,96]. Distinguishing the roles of ROS during abiotic and biotic stress can be challenging. Here, we focus on ROS wall physiology under abiotic stress and will not address the roles of ROS in responses to biotic stress.

ROS are produced by different cellular compartments, each having its own complement of ROS producing enzymes to generate a specific redox signature [94]. Membranes were initially thought to act as barriers for ROS, however, H_2_O_2_ has been shown to move across membranes to different cellular compartments through aquaporin mediated transport [97]. The presence of ROS in the wall has been studied using microscopic methods, such as imaging deposits of cerium-peroxide under transmission electron microscope and imaging ROS-reactive fluorescent probes [98,99]. Alternatively, the actual levels of apoplastic ROS have been measured either directly or indirectly by antioxidant enzyme activity assays. Under physiological conditions apoplastic ROS concentrations are approximately 10–25 pmol g^−1^ and this increases up to four times under stress conditions, together with increased activity of antioxidant enzymes [87,100]. ROS in the apoplast initiates changes in pH, protein phosphorylation status, immobilization of proteins [101,102,103,104], changes in protein structure through disulfide bond formation [105,106], and either polysaccharide cross-linking or chain scission [107] (Figure 1).

### 4.1. Wall ROS Production and Signaling

The production of ROS in the wall occurs by at least four different mechanisms. The best studied are plasma membrane (PM)-bound NADPH-oxidase Respiratory Blast Oxidase Homologs (RBOH) due to their importance for wall-mediated abiotic stress responses. RBOHs can be activated by influx of apoplastic Ca^2+^ into the cell, likely as a result of mechanical stress [94,108]. RBOH produces apoplastic superoxide which further activates Ca^2+^ influx into neighboring cells, creating a systemic response throughout the tissue which results in stress acclimatization [25]. Superoxide is further converted into H_2_O_2_ by wall SODs (Figure 1). Other proposed mechanisms for ROS production in walls during abiotic stress are via xanthine dehydrogenases [109] and wall peroxidases [110]. Oxalate peroxidase was also shown to contribute to the pool of apoplastic H_2_O_2_ and participate in acclimation of roots to drought stress [111]. Mechanisms for detoxification of wall ROS mainly include the wall peroxidases CuZn-SOD and APX, as well as low levels of ascorbate and glutathione [112]. It is important to note that these ROS detoxification systems are less effective than those present within the cell. This enables uninterrupted production of ROS in walls during stress conditions to facilitate ROS-mediated signaling and plant acclimatization to stress.

Plant hormones are also known to play an important role in ROS production. Hormone signaling pathways are involved in ROS production during both normal conditions [113] and under abiotic stress [93,94]. However, the exact mechanisms by which hormones induce ROS production during abiotic stress remain to be described. Auxin, a major plant hormone, can initiate production of ROS, which in turn alters the balance of conjugated and active auxin. This results in a negative feedback loop and impacts on plant growth [114]. The exact machinery by which auxin mediates production of apoplastic ROS remains unknown. One possible mechanism is that auxin could modulate Rho-GTPases (RACs/ROPs) which are known to interact with RBOH, resulting in increased levels of wall associated ROS [115]. It is proposed that auxin-ROS cross-talk plays an important role in *A. thaliana* tolerance to cadmium and cooper stress [116]. Brassinosteroids (BRs) are also known to induce production of ROS and have been shown to be important in ROS mediated resistance to heat stress in tomato [117]. BRs are associated with abiotic stress-related signaling networks as one of their many roles in biochemical and physiological processes [118]. Exogenous application of the BR 24-epibrassinolide induced transcription of *RBOH* and an increase in RBOH activity and apoplastic H_2_O_2_ concentration. Silencing of *RBOH1* and *Mitogen Activate Protein Kinase 2* genes in tomato abolished BR-induced increases in H_2_O_2_ [117]. Abscisic acid (ABA) has also been shown to have a similar effect on production of apoplastic H_2_O_2_ through regulating *RBOH* expression. It has been speculated that BRs and ABA function in a positive feedback loop, whereby BRs induce RBOH activation and production of H_2_O_2_ which in turn stimulates production of ABA. This could lead to prolonged production of wall H_2_O_2_ and longer stress tolerance [119].

Although it is established that ROS interact with Ca^2+^ channels in the PM, important questions remain as to how apoplastic ROS are being sensed by the cell. Do ROS receptors exist and if so are they present in the wall, the PM, or both? The existence of proteins acting as direct ROS receptors has been proposed [120], for example, oxidation of the apoplastic region of receptor-like kinases (RLKs) by ROS results in modulation of intracellular signal pathways [121]. ROS has been shown to oxidize polyunsaturated fatty acids in the PM, interact with CWI sensors, innate immunity receptors, and heterotrimeric PM-bound G-proteins [121]. Detection of ROS and induction of wall changes is therefore complex and likely to be mediated by numerous pathways. Although it is well-established that ROS production is linked with both CWI sensing and hormone signaling pathways, the exact mechanisms by which different plant hormones initiate ROS production during abiotic stress, remains to be discovered.

### 4.2. Role of Peroxidases in Wall Remodeling

ROS-induced activity of peroxidases is the primary mechanism involved in wall remodeling during stress. Peroxidases are proposed to crosslink wall glycoproteins such as extensins, facilitate arabinoxylan cross-linking through ferulic acid in grass species, and promote formation of diferulic acid cross-links between lignin molecules [122,123]. To perform their wall remodeling role, peroxidases require H_2_O_2_ as a co-substrate. Increases in H_2_O_2_ and peroxidases seems to be concurrent in osmotic stress conditions with RBOH converting superoxide to H_2_O_2_ to provide enough “fuel” for peroxidases [20]. H_2_O_2_ production during stress commonly results in wall stiffening and reduced cellular elongation due to peroxidase catalyzed cross-linking of wall components [124] (Figure 1). The resulting increase in mechanical strength is proposed to enable plant cells to endure changes in turgor during the osmotic stress caused by drought, frost, or high salinity and avoid cell damage. Increased mechanical strength of the wall also causes cessation of cell expansion. Peroxidases can also cause wall loosening. Studies have shown that the highly reactive OH^–^ (hydroxyl) radical can cleave polysaccharide glycosidic bonds without enzymatic catalysis, thus effectively reducing wall stiffness [125,126,127]. ROS-mediated wall loosening likely plays an important role during salt, drought, and heat stress to maintain cell growth even in cases of variable turgor pressure. Hydroxyl radicals are produced from H_2_O_2_ and O_2_^●−^ by peroxidases in Fenton-type reactions [124]. Which of these two mechanisms will prevail depends on biochemical parameters of the wall itself. Different studies using the same abiotic stress, for example either drought or high salinity, have shown different responses, with the wall becoming stiffer in some cases and looser in others [32]. The balance between wall stiffening or loosening will be influenced by peroxidase activity, different wall components available as substrates for peroxidases and the types of ROS produced during the stress. Walls will become stiffer and the overall mechanical stability of tissues and cells will increase if stress causes an increase of wall peroxidases activity, increase in H_2_O_2_ concentration and/or an excess of peroxidase substrates [125]. However, if peroxidase activity is decreased or substrates levels are low, production of hydroxyl radicals will prevail, which will lead to polysaccharide cleavage and a decrease of wall stiffness [125]. Why the same conditions can have different effects on wall mechanical properties is still poorly understood and requires further investigation in order to better understand the effect(s) that abiotic stresses have on wall structure and how these changes influence plant acclimatization to abiotic stress.

Peroxidases are also involved in ectopic deposition of lignin, which is one of the common responses of cells to both abiotic and biotic stress. It has been shown that during root development in rice increased transcript levels of *PHENYLALANINE AMMONIA LYASE* (*PAL*) were correlated with increased levels of wall peroxidases and the corresponding enzyme activities [122]. PAL converts L-phenylalanine into *trans*-cinnamate, which is the first step in the phenylpropanoid biosynthetic pathway, and the first step in lignin biosynthesis. This suggests that cooperative activity of both PAL and apoplastic peroxidases is important in both the production and crosslinking of phenolic compounds such as ferulic acid monomers in the wall [122]. Apoplastic peroxidase-mediated cross-linking of ferulic acid in shoots of corn plants grown under high salinity induces growth suppression in the initial phase of salt stress in salt resistant cultivars. This causes very fast oxidative irreversible fixation of shape and arrest of leaf growth. Plants also exhibited an increase in arabinose, the sugar to which ferulic acid is bound within arabinoxylans of the wall [50]. Partial or complete arrest of shoot growth is proposed to prevent excessive loss or water and to preserve energy for strategies used to tolerate salt toxicity.

A role for peroxidases in drought stress tolerance is also supported by studies of cotton roots from two drought tolerant cultivars which showed increased peroxidase transcript levels when compared to drought-sensitive lines [41]. However, in some cases the transcriptional increase in peroxidases may be misleading. While it has been determined that drought tolerant cultivars of wheat exhibit higher expression of wall peroxidase genes (*TaPRX01*, *TaPRX03*, and *TaPRX04*) [128], the in vivo activity of peroxidases was shown to be higher in drought-sensitive cultivars. Further work is required to determine the complex regulatory mechanism(s) of peroxidase activity which can be influenced by the supply of both wall substrates and H_2_O_2_, and the levels of peroxidase-modulating agents [129].

Peroxidases are also suggested to function in response to wall damage. Expression of *Arabidopsis thaliana PEROXIDASE 71* (*AtPRX71*) was increased in the pectin deficient *quasimodo 2-1* (*qua2-1*) mutants which are characterized by severely decreased growth and perturbed morphology. Double knockout *qua2-1 atprx 71-2* mutants have phenotypes similar to wild-type (WT) suggesting PRX71 activity makes a significant contribution to the *qua2-1* growth defects [130]. These findings support the hypothesis that ROS-mediated wall remodeling is triggered by a loss of CWI. Ectopic deposition of lignin during stress is generally accepted as a compensatory mechanism to wall damage and loss of wall integrity. For example, disruption of cellulose synthesis by the drug isoxaben induces wall damage and was shown to result in ectopic lignification in *A. thaliana* root cells [131]. *A. thaliana* plants transformed with *Potentilla astrosanguinea SOD* and *Rheum australe APX* resulted in increased lignin deposition in vascular tissues when exposed to osmotic stress. This stress-induced lignin deposition is proposed to enable vascular tissues to withstand changes in osmotic pressure associated with salt stress [132]. The coordinated action of PaSOD and RaAPX in xylem was observed, where PaSOD produced enough H_2_O_2_ to facilitate lignin polymerization as well as initiate signaling cascades to increase expression of *no apical meristem*, *ATAF and cup shaped* (*NAC*), *myeloblastoma* (*MYB*), and *WRKY* domain-containing transcription factors that regulate genes involved in stress responses, while RaAPX maintained optimum H_2_O_2_ levels [132].

Thus, ROS and the entire redox system have extremely important roles in wall-mediated responses to abiotic stress in plants (Figure 1). The generation of ROS signals is well-characterized whereas how signaling ROS are perceived and translated into stress responses is extremely diverse and largely depends on the ROS-induced modification of target proteins and wall polysaccharides. It also remains to be established exactly how ROS signaling networks are fine tuned. Further understanding of the pathways with which ROS interacts, such as CWI sensors, will aid in elucidating its complex functions.

## 5. Cell Wall Integrity as a Measure of Stress

Wall mechanical integrity is constantly monitored by CWI sensors [33]. Most of the characterized CWI sensors belong to the RLK superfamily. RLKs consist of an extracellular domain, single transmembrane region, and intracellular kinase. RLKs are a hugely diverse gene family with more than 610 members identified in *A. thaliana* and over 1130 in rice [35]. Wall RLKs are important components of signaling pathways involved in development, growth, reproduction, and response to abiotic and biotic stress. A subset of CWI sensors has been shown to monitor wall status and initiate different cellular responses to wall structural changes, including those induced by stress conditions. A number of recent papers have extensively reviewed CWI sensing [33,34]. Here, we provide a summary of the best characterized CWI sensing RLKs and other CWI sensors likely to have roles in abiotic stress-induced wall remodeling (Figure 1).

### 5.1. Wall-Associated Kinases (WAKs)

The WAKs are a subset of RLKs with roles in cell expansion and response to different stress conditions such as high soil salinity and increased concentrations of heavy metals [133,134,135]. For example, *AtWAK1* expression in *A. thaliana* roots was shown to be linked to increased aluminium levels in soil [135], and the expression of *OsWAK11* in rice was increased by aluminium and copper [136]. WAKs are characterized by extracellular domains with motifs similar to vertebrate epidermal growth factor repeats. Strong support for WAKs acting as CWI sensors lies in the fact that some WAKs form covalent bonds with pectins; for AtWAK1 and AtWAK2 this was shown both in vivo and in vitro [137]. In in vitro assays, WAKs have shown more affinity for de-esterified rather than esterified pectins and can also bind oligogalacturonides (OG) and rhamnogalactouronans I and II [137]. WAK binding to de-esterified pectins is proposed to activate signal transduction pathways to promote cell expansion, whereas OG binding activates stress responses. Knockout mutants of *atwak2* in *A. thaliana* displayed reduced root growth compared to WT and a reduction in levels of vacuolar invertases [138]. Vacuolar invertases contribute to the regulation of solute levels and therefore turgor maintenance by the hydrolysis of sucrose to glucose and fructose [139]. Replacement of the extracellular domain of WAK2 with that of the BR receptor BRI1 [140] and expression of BRI-WAK2 in protoplasts treated with brassinolide resulted in reduced shrinking of protoplasts and increased survival, supporting the indirect regulation of solute concentration and turgor by WAK2 [138]. Interestingly, a dominant allele of WAK2 induces stress responses [141] suggesting WAKs monitor the requirement for either expansion or stress responses. This is proposed to be through competitive binding of either OG or native pectin and activation of different MAPK-dependent signaling pathways [141].

### 5.2. CWI Sensors from Catharanthus roseus Receptor-Like Kinases (CrRLK) Family

A number of CWI sensors from the CrRLK family have been well-characterized. THESEUS1 (THE1) was discovered in a mutant screen for suppressors of the increased hypocotyl length phenotype of the *CELLULOSE SYNTHASE A*6 (*CESA6*)-deficient mutant *procuste1-1* (*prc1-1*) [66]. Double *the1 prc1-1* mutants exhibited intermediate phenotypes between WT and *prc1-1*, whereas overexpression of *THE1* in WT did not show any phenotype. Overexpression of *THE1* in the *prc1-1* background resulted in ectopic lignin deposition, suggesting THE1 could be participating in mechanically reinforcing the weakened cellulose-deficient stem of *prc1-1*. These observations led to the conclusion that THE1 acts as a CWI sensor which is activated only upon stress-induced wall perturbation. Wall fragments or secondary messengers formed during stress conditions could act as ligands for THE1 [66]. Recently, a possible ligand for RAPID ALKALINIZATION FACTOR 34 (RALF34), which is important for THE1-mediated CWI sensing, has been identified [64]. The RALF34 ligand is necessary for lateral root formation, however the signaling pathway triggered by RALF34 remains to be elucidated.

Another CrRLK1 member, FERONIA (FER), was first described as a protein important for CWI sensing during pollen tube growth. FER is essential for successful rupture of the wall at the pollen tube tip and discharge of spermatic nuclei in the embryo sac of *A. thaliana* [142,143]. The extracellular tandem domains of FER show homology to the animal protein malectin, which binds oligosaccharide fragments of glycosaminoglycans and is important for protein quality control in the endoplasmic reticulum [144]. Like WAKs, it is thought that FER binds pectin via its malectin-like A and B domains. FER has been shown to be involved in salinity tolerance [65] and is proposed to sense changes in pectin structure and wall elasticity to activate signaling pathways [65]. FER was shown to initiate cell-specific transient Ca^2+^ influxes in response to salt stress, followed by wall remodeling and repair, and restoration of cell growth [65]. Downstream components of FER signaling, as well as the intracellular targets of Ca^2+^, remain unknown. FER also directly interacts with signaling ROS production. FER interaction with RAC/ROPs enables GTP-dependent activation of RBOH [115], initiating ROS production and signaling cascades as discussed in Section 3.

While FER is associated with sensing pectin damage in the wall, other RLKs are proposed to monitor the integrity of cellulose. The LEUCINE-RICH REPEAT (LRR)-RLK, MALE DISCOVERER 1-INTERACTING RLK 2/LRR-KINASE FAMILY PROTEIN INDUCED BY SALT STRESS (MIK2/LRR-KISS), was shown to be an important sensor of cellulose integrity in the wall. Phenotypes of *mik2/lrr-kiss* mutants include increased sensitivity to cellulose synthesis inhibitors, decreased expression of pathogen immunity genes, reduced levels of jasmonate, and decreased lignin deposition [145]. The attenuated CWI responses in *mik2/lrr-kiss* mutants suggest MIK2/LRR-KISS acts in pathways to mechanically reinforce compromised walls during abiotic stress and/or pathogen attack. MIK2/LRR-KISS could either directly detect mechanical changes in CWI or detect signals generated by wall damage. *mik2/lrr-kiss* mutants also showed increased sensitivity to salt stress, which could be explained by diminished cellulose-sensing capacity [145].

Although numerous *CrRLK1* family CWI sensors have been characterized since the initial discovery of THE1 and FER, the exact signaling pathways and intercellular targets of these important proteins remain unknown. Further elucidation of abiotic stress-induced wall remodeling will be possible only when the signaling networks of the different CWI sensors are fully understood.

### 5.3. Fasciclin-Like Arabinogalactan-Proteins (FLAs)

RLKs commonly form hetero- and/or homodimers [146,147] and can be part of larger complexes [148]. Other types of putative CWI sensors could form part of these complexes and be directly or indirectly involved in stress-related wall remodeling. The arabinogalactan-protein (AGP) family of glycoproteins is a good candidate for CWI sensors as these glycoproteins are closely associated with the wall and have been implicated in both development and stress-related functions. AGPs are characterized by type II arabinogalactan chains (5–25 kD) *O*-linked to hydroxyproline (Hyp) residues in the protein backbone [149]. Another distinguishing feature of many AGPs is the presence of a glycosylphosphatidylinositol (GPI) anchor that attaches AGPs to the PM [149].

A subgroup of AGPs, the Fasciclin-like AGPs (FLAs), have gained particular interest due to their numerous roles in plant development including: pollen microspore development, seed coat mucilage production, cell expansion, stem biomechanics, and initiation and elongation of cotton fibers [150,151,152,153]. FLAs are characterized by the presence of fasciclin-1 (FAS1)-like domains in addition to the AGP glycomotifs [154]. FAS1 domain containing proteins have largely been characterized in animal model systems as being important for cell–cell communication and interactions with the extracellular matrix (ECM) [155,156]. Plant FLAs are also proposed to interact with wall polysaccharides and CWI sensors suggesting they play comparable roles to that of animal FAS1 proteins. A role in stress responses is supported by phenotypes of a number of *fla* mutants, including *salt overly sensitive5* (*sos5*) which revealed a role for FLA4/SOS5 in CWI during salt stress [150] and *fla9* mutants which show an increased seed abortion phenotype during drought stress [157].

FLA4/SOS5 has also been shown to play a role in seed coat mucilage adherence and organization [158]. Genetic studies suggest FLA4/SOS5 acts in the same pathway as the LRR-RLKs FEI1 and FEI2. Loss-of-function mutants for these genes show reduced seed mucilage adherence and this is proposed to be due to changes in pectin interactions [159]. Although it has not been shown specifically for FLAs, AG-glycans have been shown to cross-link with pectins in APAP1 [160]. Potentially, changes in pectin integrity could be transmitted via FLA4 to FEI1/2 to initiate signaling in response to abiotic stress [161].

A different subset of FLAs has a role in maintaining the biomechanical properties of stems and this role is conserved in several species [96,102]. For example, *Populus trichocarpa PtFLA6* has been shown to play an important role in stress-induced wall remodeling during tension wood formation [161]. Woody plants are often subjected to considerable mechanical stress from environmental conditions, such as wind, snow, or growth on a slope. In response, some woody plants develop tension wood on the upper side of those organs under constant bending or leaning to pull the branch or stem to its original position or provide mechanical strength to endure the stress [162]. PtFLA6 was shown to participate in tension wood formation through gibberellin (GA)-mediated signaling pathways [163]. *PtFLA6* is upregulated during tension wood formation and reduced levels of *PtFLA6* using antisense RNA resulted in less tension wood production in *P. trichocarpa* [163]. Inhibitors of GA synthesis caused decreased degradation of the poplar DELLA protein PtRGA1, which coincided with lower expression of *PtFLA6* [163]. In contrast, application of GA_3_ promoted *PtFLA6* expression. In response to mechanical stress GA production is proposed to lead to degradation of DELLA proteins such as RGA1, resulting in *PtFLA6* expression and formation of tension wood [163].

How FLAs sense mechanical changes in the wall and initiate signaling is currently unclear. The potential of FLAs to make multiple interactions, through FAS1-like domains and/or glycans as well as the potential to localize to lipid microdomains through their GPI-anchor, suggest they may form part of CWI sensing complexes. Identification of interacting proteins and/or polysaccharides of FLAs is needed to determine their roles in detection of abiotic stress and stress-induced wall remodeling. The signal transduction mechanism(s) and signaling pathways downstream of FLAs are also unknown and require elucidation.

### 5.4. Mechanosensitive Ion Channels

Mechanosensitive (MS) channels at the PM are shown to be involved in the movement of ions in response to membrane tension [164]. Numerous studies, although not identifying direct effectors, have shown the importance of mechanically-dependent Ca^2+^ influxes in wall remodeling and cell growth under physiological conditions, which could also imply that MS channels participate in stress-induced wall remodeling. For example, in tip growing cells such as root hairs of *A. thaliana* and pollen tubes of *Lilium longiflorum*, mechanical deformation of the PM was followed by a fast transient Ca^2+^ influx [165], which leads to RBOH activation and production of wall ROS [166].

Two of the best characterized families of MS ion channels in plants are MECHANOSENSITIVE CHANNELS OF SMALL CONDUCTANCE-LIKE (MSL) AND MATING PHEROMONE INDUCED DEATH 1 COMPLEMENTING ACTIVITY (MCA) [61]. MCA1 and 2 produce increased Ca^2+^ currents in cultured cells of *Nicotiana tabacum* and *Oryza sativa* as a response to increased cell swelling, protecting cells from hypo-osmotic shock. MCA1 is also important for *A. thaliana* roots penetration through agar, suggesting it could have a role in mechano-sensing during root growth [167,168,169]. MSL8 has been shown to conduct Cl^−^ ions and *atmsl8* mutants in *A. thaliana* show defects in pollen grain germination. MSL10 is involved in ROS-induced cell death in *A. thaliana* cells independently of its ion trafficking function [170,171]. A recent study has shown the importance of MCA and MSL channels in response to isoxaben-induced wall damage. Ectopic lignin deposition caused by wall damage was reduced in *mca1* mutants and enhanced in quintuple *msl4msl5msl6msl9msl10* mutants. This study also showed that MCA1 and MSL2 and 3 have roles in the production of jasmonic acid (JA), a plant hormone important during stress-induced wall remodeling, as *mca1*, *msl2*, and *msl3* produced less jasmonate in response to isoxaben-induced wall damage [55].

Another interesting MS protein is DEFECTIVE KERNEL 1 (DEK1). DEK1 is a plant-specific member of the calpain superfamily of Ca^2+^-activated Cys-proteases [172]. Electrophysiological studies have shown that a mechanically-mediated Ca^2+^ influx is dependent on DEK1 activity [173]. Studies have shown that DEK1 affects numerous aspects of plant development likely via regulation of wall composition and thickness [172,174,175,176]. Models have been proposed that suggest DEK1 is activated by mechanical signals at the PM and initiates signaling cascades to regulate cell wall-related genes and ensure CWI [176]. Although clearly important during normal growth and development, further studies are required to determine if DEK1 plays a role in abiotic stress-induced wall remodeling.

Although CWI sensors (CrRLKs, WAKs, FLAs, and others) and MS channels are involved in abiotic stress-induced wall remodeling, it remains unclear if they act independently or there is a cross talk between these groups of proteins. Detection and production of ROS coupled with CWI sensing and mechanosensing at the PM are important components of the abiotic stress detection and wall remodeling machinery. However, they are actively interacting with a third component of this system, plant hormones. In most cases plant hormones regulate stress-induced wall remodeling indirectly, by controlling gene expression of different protein sensors and receptors and wall remodeling enzymes.

## 6. Hormone Signaling in Response to Cell Wall Damage

Plant hormones have broadly been defined in terms of those having roles in growth and development, such as auxins, cytokinins, ethylene, and GAs, and those considered to be “stress” hormones, such as brassinosteroids (BR), JA, salicylic acid (SA), ABA, and others [177]. However, hormone signaling pathways are extremely diverse and many hormones participate both in development and stress responses [163,178]. Increasingly the role of hormones such as ABA and GA are being implicated in abiotic stress pathways [179]. Here, we discuss the hormones BR and JA which have been directly implicated in abiotic stress-induced wall remodeling and shown to intersect with CWI and ROS pathways.

Abiotic stress-induced cellulose damage and/or reduction results in increased JA, SA, and ethylene levels, changes in wall composition and structure, and ectopic deposition of lignin [180,181,182,183,184]. Surprisingly, some studies have shown that JA can act as an inhibitor of ectopic lignin production during stress-induced wall remodeling. *A. thaliana* mutants unable to produce JA, such as *allene oxide synthase* (*aos*) and *jasmonic acid resistant 1* (*jar1*), showed increased levels of wall damage-induced synthesis of lignin [52]. In contrast, mutants impaired in ROS production and CWI detection, such as *rboh* and *the1* mutants, have increased levels of ectopic wall damage-induced lignin. This led to the conclusion that interactions between the JA, ROS, and CWI pathways are involved in responses to cell wall damage [52].

There is also the possibility that some MS channels in *A. thaliana*, such as MSL2 and MSL3, as well as MCA1 could be coordinated with JA signaling to regulate stress-induced lignin deposition in *A. thaliana* [55]. Overexpression of the JA receptor, CORONATINE INSENSITIVE 1 (COI1) in *A. thaliana* cell suspension cultures resulted in elevated expression of genes encoding the wall remodeling enzymes OLIGOGALACTURONIDE OXIDASE 1, β-GLUCOSIDASE, ENDOGLUCANASES, and POLYGALACTURONASE INHIBITING PROTEIN 2 [185]. These findings reinforce the role of hormone-mediated wall remodeling during abiotic stress as part of a complex system involving mechano-sensing and ROS production.

BRs are also known to be involved in responses to wall damage and genes encoding the wall loosening XTHs and expansins are upregulated by BR [186,187,188]. Cellulose production in *A. thaliana* is regulated via BR signaling, modulating CESAs at both transcriptional and post-transcriptional levels. The transcription factor BES1 is activated through BR signaling and can directly bind to CANNTG-E motifs in the promoters of *CESA* genes [189]. In addition, the kinase BRASSINOSTEROID INSENSITIVE 2 (BIN2), a negative regulator of BR signaling, can phosphorylate CESA1 [190]. BR regulation of cellulose during stress appears to be evolutionarily conserved as when subjected to drought stress, wild wheat, *Agropyron elongatum*, showed elevated levels of *CESA3* and genes involved in BR signaling. This could partly explain the higher drought-tolerance of wild genotypes of *A. elongatum*, which developed larger root and shoot biomass under drought conditions when compared to domesticated genotypes [191]. There is some evidence that BR does not directly regulate cellulose content, rather it regulates cortical microtubule positioning and organization, which in turn determine the organization of cellulose microfibrils in wall [192,193]. Further work is needed to clarify the relationship between BR and cellulose synthesis during stress.

BRs are also known to be involved in stress-induced remodeling of pectins. Cold stress-induced increases in *AtPME41* transcript levels and corresponding enzyme activity was shown to be BR-dependent and led to cold stress-induced wall stiffening [194]. In *A. thaliana* BR signaling was shown to contribute to the regulation of wall modifying proteins, including PMEs [195]. BR involvement in a stress-induced feedback mechanism from the wall was shown by a mutation in the BR receptor BRI1 being able to overcome the growth inhibitory effects of a PMEI overexpression line [81,195]. The connection between CWI sensing and BR signaling in *A. thaliana* was shown to involve a PM-localized receptor-like protein RLP44 that can directly interact with the BR receptor kinase BAK1 [81] (Figure 1).

During both physiological conditions and abiotic stress BRs are known to regulate monolignol biosynthesis and also potentially influence polymerization of monolignols and phenolic acids [196]. Application of exogenous BR resulted in considerably increased expression of genes (*APX*, *CAT*, *SOD*, and *PRX*) encoding the redox enzymes in maize, rice, and wheat grown under heavy metal stress and elevation of the corresponding protein activity [196,197]. Peroxidase catalyzed production of phenolic radicals was shown to enable lignin polymerization and crosslinking of ferulic acid esterified to arabinoxylans, the latter being highly characteristic of grasses [198]. Hormones are actively involved in abiotic stress responses and the BR and JA signaling pathways are clearly shown to overlap with ROS production and CWI maintenance systems (Figure 1).

## 7. Future Perspectives

**Figure 2:**
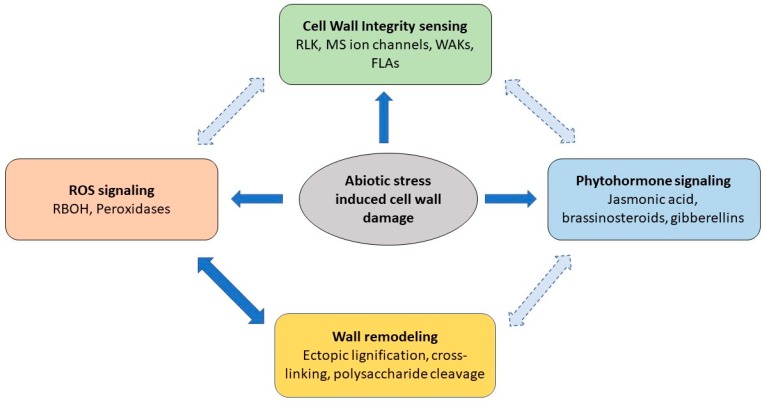
Abiotic stress-induced wall sensing and signaling is coordinated through multiple pathways to ensure precise changes to the wall and ensure mechanical integrity. The exact mechanisms by which ROS production, CWI sensing, and hormone signaling pathways are interconnected remain to be discovered and likely involve direct (solid arrow) and indirect (dashed arrow) interactions.

The importance of ROS, wall-associated proteins, CWI sensors, and hormones during abiotic stress signaling is unequivocal. We propose that these different elements of wall sensing, signaling, and wall synthesis machinery are interconnected and fine-tuned in response to abiotic stress to increase chances of plant survival (Figure 2). However, significant gaps remain in characterizing the downstream targets and complex interactions between the components of stress response systems (Figure 2). This is not trivial given the complex interacting networks function during both normal growth and development as well as being switched on during stress responses. How the levels of hormones and ROS are fine-tuned will be crucial to our understanding of stress responses.

Arguably, the most significant challenge in studying abiotic stress-induced cell wall remodeling is understanding the complex CWI and mechanosensor signaling networks. Although there has been considerable progress in the last decade, we are only just beginning to understand how CWI sensors are detecting changes in the cell wall caused by abiotic stresses. The intracellular elements of the signaling networks are also largely undefined. To further add to the layers of complexity, different MS proteins, such as MSL and MSA proteins, also interact with CWI sensing, and both MS and CWI activity is connected with hormone signaling and ROS production. To fully understand abiotic stress-mediated wall remodeling, we also need to understand how plants can differentiate between physiological and stress conditions, switch on and off different components of this network, and how these signals are integrated after perception to generate a relatively narrow span of targeted responses, such as change in ROS production, peroxidase activity, and other wall remodeling enzymes activity.

To date, studies have largely focused on the transcriptional and proteomic changes as a means of understanding the roles that walls play in plant responses to abiotic stress. These approaches need to be complemented with biochemical and cell biological research to further elucidate signaling pathways and networks which are involved in wall-mediated stress responses. Single-cell transcriptomic and metabolomic approaches would also provide enhanced resolution of regulatory processes. Advances in tissue imaging techniques such as spinning disc confocal microscopy and atomic force microscopy are proving powerful methods to visualize how components of the ROS, CWI, and hormone pathways regulate the wall [49,57] and how remodeling affects wall ultrastructure, organization, and composition [57]. Combined with the enhanced sensitivity of MS-based analytical techniques these have given insight into how stress-related remodeling affects the chemical composition of the wall. Recently developed synthetic glycan microarrays are also emerging as a powerful technique to characterize wall polysaccharide directed antibodies that are crucial in visualizing changes in the organization/structure of wall polysaccharides during normal growth and in response to stresses [199]. Defining the cellular- and tissue-level specificity of wall polysaccharides will aid the ability to link wall structural changes with physical properties and function during abiotic stress. Stress responses in dicot models, such as *A. thaliana*, remain the best characterized systems. Walls of the commercially important commelinid monocots, that include the cereals (wheat/barley/rice/corn), differ from those of most dicots and justify a similar depth of investigation of stress response pathways if we are to sustain grain production in a resource constrained world with an ever-growing population. By better understanding the role of the wall to modulate plant growth during stress we can begin to develop strategies towards improving plant resilience and, hence, productivity.
